# A novel negevirus isolated from *Aedes vexans* mosquitoes in Finland

**DOI:** 10.1007/s00705-020-04810-4

**Published:** 2020-09-20

**Authors:** Maija T. Suvanto, Phuoc Truong Nguyen, Ruut Uusitalo, Essi M. Korhonen, Giulia Faolotto, Olli Vapalahti, Eili Huhtamo, Teemu Smura

**Affiliations:** 1 Department of Virology, Medicum, University of Helsinki, Helsinki, Finland; 2 Department of Veterinary Biosciences, University of Helsinki, Helsinki, Finland; 3 Department of Geosciences and Geography, University of Helsinki, Helsinki, Finland; 4 Laboratory of Microbiology and Virology, University Hospital Maggiore Della Carità Di Novara, Piemonte, Novara, Italy; 5 Department of Virology and Immunology, Helsinki University Hospital, HUSLAB, Helsinki, Finland

## Abstract

Negeviruses are insect-specific enveloped RNA viruses that have been detected in mosquitoes and sandflies from various geographical locations. Here, we describe a new negevirus from Northern Europe, isolated from pool of *Aedes vexans* mosquitoes collected in Finland, designated as Mekrijärvi negevirus (MEJNV). MEJNV had a typical negevirus genome organization, is 9,740 nucleotides in length, and has a GC content of 47.53%. The MEJNV genome contains three ORFs, each containing the following identified conserved domains: ORF1 (7,068 nt) encodes a viral methyltransferase, an FtsJ-like methyltransferase, a viral RNA helicase, and an RNA-dependent RNA polymerase, ORF2 (1,242 nt) encodes a putative virion glycoprotein, and ORF3 (660 nt) encodes a putative virion membrane protein. A distinctive feature relative to other currently known negeviruses is a 7-nucleotide-long overlap between ORF1 and ORF2. MEJNV shares the highest sequence identity with Ying Kou virus from China, with 67.71% nucleotide and 75.19% and 59.00% amino acid sequence identity in ORF 1 and ORF 2, respectively. ORF3 had the highest amino acid sequence similarity to Daeseongdong virus 1 and negevirus Nona 1, both with 77.61% identity, and to Ying Kou virus, with 71.22% identity. MEJNV is currently the northernmost negevirus described. Our report supports the view that negeviruses are a globally distributed, diverse group of viruses that can be found from mosquitoes in a wide range of terrestrial biomes from tropical to boreal forests.

Negeviruses are positive-sense single-stranded enveloped RNA viruses of insects. They have been isolated from mosquitoes of various genera (e.g., *Culex, Aedes, Anopheles,* and *Psorophora*) and from *Lutzomyia* sandflies around the world, including the Americas, Africa, Middle East, Europe, Asia, and Australia [1–6]. Negeviruses have 9- to 10-kb genomes that contain three open reading frames (ORFs). ORF1 encodes a methyltransferase, an FTsJ-like helicase, a viral RNA helicase, and an RNA-dependent RNA polymerase (RdRp), ORF2 encodes glycoproteins, and ORF3 encodes virion membrane proteins [1–3]. The recently proposed "*Negevirus*" taxon consists of two clades, named "*Nelorpivirus*" and "*Sandewavirus*" [3, 5], and the taxon is related to plant viruses of the genera *Cilevirus, Higrevirus* and *Blunervirus* [1, 7]. In recent years, the "*Negevirus*" taxon has been expanding with several new negeviruses being reported. The geographical origins of mosquito-associated negeviruses include various terrestrial biomes, mainly temperate, subtropical, and tropical forest areas. Here, we report a novel negevirus isolated from *Aedes vexans* mosquitoes from a boreal coniferous forest area in Finland.

Mosquitoes were collected for an arbovirus study in August 2018 in Mekrijärvi (62°45′43″N, 30°57′32″E), Eastern Finland [8]. One mosquito pool consisting of 26 individual female mosquitoes caused a strong cytopathic effect (CPE) in *Aedes albopictus* C6/36 cells. The voucher specimen for this mosquito pool was identified morphologically [9] as *Aedes vexans*. RNA was extracted from virus isolation supernatant collected 10 days after infection, using TRIzol Reagent (Thermo Fisher). A sequencing library was prepared using an NEBNext Ultra II RNA Library Prep Kit (New England Biolabs), quantified using an NEBNext Library Quant Kit for Illumina (New England Biolabs), and sequenced on a MiSeq platform using a MiSeq Reagent Kit v2 with 150-bp paired-end reads. Raw sequence reads were trimmed, and low-quality (quality score < 30) and short (< 50 nt) sequences were removed using Trimmomatic [10]. Thereafter, *de novo*-assembly was conducted using MegaHit, followed by re-assembly against the *de novo* assembled consensus sequence using the BWA-MEM algorithm [11] implemented in SAMTools version 1.8 [12]. A total of 357,745 reads mapped to the largest contig, with mean coverage of 7,624 (range, 146-17,546).

A genomic sequence of 9,740 nt was identified as a negevirus using an NCBI BLASTx search, and the isolate was designated as "Mekrijärvi negevirus" (MEJNV) (GenBank accession number MT522375). Three ORFs were identified in the MEJNV genome using NCBI ORFfinder (https://www.ncbi.nlm.nih.gov/orffinder/) (Fig. 1), flanked by untranslated regions (unverified) of 509 nt (5’UTR) and 162 nt (3’UTR) in length. MEJNV was most similar to Ying Kou virus strain YK1714 (NC_040636.1) originating from *Culex pipiens pallens* mosquitoes in China, with 67.71% nucleotide sequence identity. The genome size and the detected ORFs were similar, and the GC content (GC%) of 47.53% was slightly higher when compared to previously described negeviruses (avg. GC% = 41.11%). The 3’terminus of ORF1 and the 5’terminus of ORF2 were found to overlap by seven nucleotides, and this is a distinct feature of the MEJNV genome. Conserved protein domains were identified using NCBI CD-search (https://www.ncbi.nlm.nih.gov/Structure/cdd/wrpsb.cgi) (Fig. 1). The identified domains and their positions within the ORFs align with those of previously described negeviruses [1] (Fig. 1). The evolutionary relationship of MEJNV to other related negeviruses was assessed by constructing a phylogenetic tree based on ORF1 amino acid sequences of negevirus strains (*n* = 43). The database sequences were aligned using MAFFT version 7.453 [13] and used to compute a midpoint-rooted maximum-likelihood tree with 1,000 bootstrap replicates in IQ-TREE version 1.6.12 [14], using the ModelFinder [15] and ultrafast bootstrap [16] algorithms (Fig. 2). ModelFinder suggested LG+F+R10 as the optimal substitution model. The final tree was visualized with FigTree version 1.4.4 (https://tree.bio.ed.ac.uk/software/figtree/). Nucleotide sequence alignments done with MAFFT showed the highest similarity between MEJNV and Ying Kou virus (NC_040636.1) from China, with 67.71% nucleotide sequence identity. Similar analysis done with amino acid sequences showed 75.19% identity between ORF1, 59.00% between ORF2, and 71.22% between ORF3 sequences. However, the closest matches to ORF3 of MEJNV were those of Daeseongdong virus 1 (NC_028487.1) and negevirus Nona 1 (AB972669.1), both with 77.61% identity. Notably, the GC% of MEJNV was similar to that of Ying Kou virus (GC% = 48.18%). Phylogenetic analysis suggested that MEJNV forms a monophyletic cluster (bootstrap value, 100) with Ying Kou virus. The closest relatives of MEJNV and Ying Kou viruses included viruses from Japan and South Korea (Daeseongdong virus 1 and negevirus Nona 1) and more distant relatives from South America, Australia, and the Philippines. Notably, the geographically closest negevirus sequence from Sweden in 2009 (Biggie virus, GenBank accession no. QGA70894) was not grouped with MEJNV. These results indicate that MEJNV is a distinct strain of negevirus related to negeviruses from distant geographical locations, and it is currently the northernmost negevirus strain to be isolated. Our results support the view that negeviruses are distributed globally in mosquitoes.

**Fig. 1:**
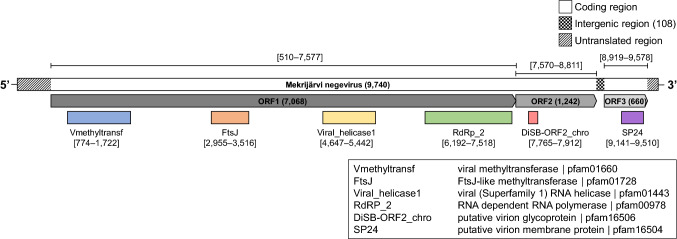
Genome organization of the novel Mekrijärvi negevirus. The lengths (in nucleotides) of ORFs 1–3 and the intergenic region are shown in parentheses. The nucleotide positions of ORFs and their putative conserved protein domains within the genome are shown in square brackets

**Fig. 2:**
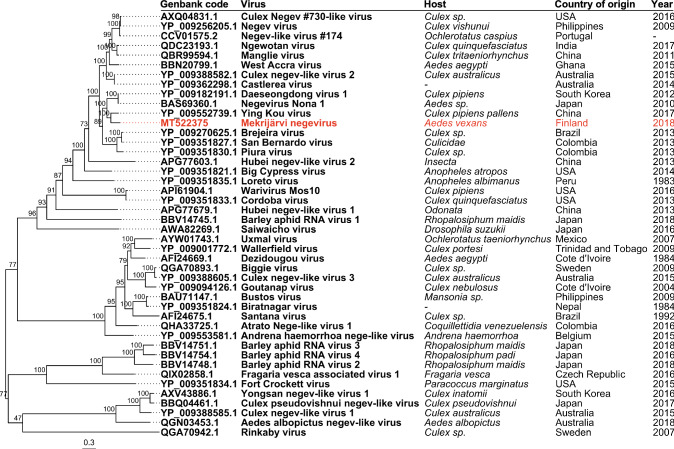
Phylogenetic tree of negeviruses and related viruses (n = 43) computed from protein sequences of ORF1 containing the RNA-dependent RNA polymerase (RdRp). The host, country of origin, and collection year are shown in square brackets. Unknown information is indicated by a hyphen. Sequences were aligned using MAFFT, and a maximum-likelihood tree with 1,000 bootstrap replicates was built using IQ-TREE. The tree is rooted at the midpoint
